# Burden of poor oral health in older age: findings from a population-based study of older British men

**DOI:** 10.1136/bmjopen-2015-009476

**Published:** 2015-12-24

**Authors:** S E Ramsay, P H Whincup, R G Watt, G Tsakos, A O Papacosta, L T Lennon, S G Wannamethee

**Affiliations:** 1 Department of Primary Care & Population Health, UCL, London, UK; 2 Population Health Research Institute, St George's University of London, London, UK; 3 Department of Epidemiology & Public Health, UCL, London, UK

**Keywords:** EPIDEMIOLOGY, ORAL MEDICINE, PUBLIC HEALTH, GERIATRIC MEDICINE

## Abstract

**Objectives:**

Evidence of the extent of poor oral health in the older UK adult population is limited. We describe the prevalence of oral health conditions, using objective clinical and subjective measures, in a population-based study of older men.

**Design:**

Cross-sectional study.

**Setting and participants:**

A representative sample of men aged 71–92 years in 2010–2012 from the British Regional Heart Study, initially recruited in 1978–1980 from general practices across Britain. Physical examination among 1660 men included the number of teeth, and periodontal disease in index teeth in each sextant (loss of attachment, periodontal pocket, gingival bleeding). Postal questionnaires (completed by 2147 men including all participants who were clinically examined) included self-rated oral health, oral impacts on daily life and current perception of dry mouth experience.

**Results:**

Among 1660 men clinically examined, 338 (20%) were edentulous and a further 728 (43%) had <21 teeth. For periodontal disease, 233 (19%) had loss of attachment (>5.5 mm) affecting 1–20% of sites while 303 (24%) had >20% sites affected. The prevalence of gingival bleeding was 16%. Among 2147 men who returned postal questionnaires, 35% reported fair/poor oral health; 11% reported difficulty eating due to oral health problems. 31% reported 1–2 symptoms of dry mouth and 20% reported 3–5 symptoms of dry mouth. The prevalence of edentulism, loss of attachment, or fair/poor self-rated oral health was greater in those from manual social class.

**Conclusions:**

These findings highlight the high burden of poor oral health in older British men. This was reflected in both the objective clinical and subjective measures of oral health conditions. The determinants of these oral health problems in older populations merit further research to reduce the burden and consequences of poor oral health in older people.

**Strengths and limitations of this study**

- This study provides novel information on the burden of oral health in a community-dwelling older British population.
- Study strengths are a socially and geographically representative sample of older British men, and the use of a range of subjective and objective clinical oral health measures.
- Limitations include limited generalisability of findings to older women and non-white ethnic groups, and lack of data on dental caries.

## Introduction

Countries such as the UK are experiencing a dramatic demographic shift, with a growing population of older people. The number of people aged ≥65 and ≥85 years in England and Wales is projected to increase by 25% and 50% respectively by 2033.^1^ These patterns of an ageing population in the UK and other countries have important public health implications,^2^ as increasing age is strongly associated with chronic disease and disability.^1^ Therefore, there is a greater imperative to prevent and manage chronic diseases and maintain optimal functioning in older people.^2^ Despite increasing research into healthy ageing and improving independence in older age, there is relatively less emphasis on oral health problems, which have a significant impact on quality of life, nutritional intake, and well-being in older age.^3^ The Global Burden of Disease 2010 Study showed that oral health problems accounted for 15 million disability-adjusted life years, implying an average health loss of 224 years per 100 000 population^4^; furthermore, due to population ageing, the burden of oral health problems increased from 1990 to 2010.^4^ This burden particularly from periodontal (gum) disease and tooth loss, increases with age.^4^ A review of the epidemiology of oral health conditions in older people highlighted the burden of conditions including tooth loss and dry mouth in older people.^5^ Although edentulism (complete loss of natural teeth) has declined in recent decades in several countries,^5^ a substantial number of older people are still edentulous (2.7 million in the UK in 2009),^6^ and partial tooth loss remains an important problem affecting eating and quality of life of older people.

In order to effectively address the issue of poor oral health in older people, it is important to understand the extent of the problem. There are few population-based epidemiological studies of older people in the UK that describe the burden of oral health problems and needs in later life. Most evidence is from studies or surveys that are not specific to older people, such as the Adult Dental Health Survey,^7^ or which have limited information on oral health, such as self-report of having natural teeth, or self-rated oral health as in the English Longitudinal Study of Ageing.^7^ ^8^ The National Diet and Nutrition Survey in 1994–1995 was the last study specifically of older people in Britain (aged >65 years) that included an oral health examination.^9^ An improved understanding of the burden of oral health problems in the UK is urgently needed in people aged over 70 years in order to understand the health needs of this growing population. This aim of this study was to describe the burden of poor oral health in a representative population-based sample of community-dwelling British men aged 71–92 years. We describe a range of oral health conditions based on both objective clinical measures, such as number of teeth, periodontal disease, oral inflammation, and also subjective measures such as self-rated oral health, impact of oral health on daily activities and dry mouth—these assessments capture a range of dental diseases and oral health conditions that are particularly important in older age.

## Methods

The British Regional Heart Study (BRHS) is a prospective study of a socially and geographically representative sample of 7735 men, aged 40–59 years from one general practice in each of 24 towns representing all major British regions, were initially examined in 1978–1980.^10^ In 2010–2012, all surviving men (n=3137, 41%) aged 71–92 years were invited to attend a 30-year re-examination.^11^ Ethical approval was provided by all relevant local research ethics committees. All participating men provided written informed consent for the investigations, which were carried out in accordance with the Declaration of Helsinki. Participants underwent a physical examination and completed a questionnaire (at the time of examination or sent by post if they did not attend the examination) providing information on their medical history and lifestyle factors. Occupational social class was based on the longest held occupation recorded at study entry (aged 40–59 years) and comprised six social class groups—I (professionals, eg, physicians, engineers), II (managerial, eg, teachers, sales managers), III non-manual (semiskilled non-manual, eg, clerks, shop assistants), III manual (semiskilled manual, eg, bricklayers), IV (partly skilled, eg, postmen) and V (unskilled, eg, porters, general labourers). For the purposes of this study, social classes III manual, IV and V were grouped as manual social class and those from the Armed Forces were not included (n=63).

The physical examination of participants in 2010–2012, at age 71–92 years, included for the first time a brief oral health assessment. Dental measures included a count of the number of teeth, and three measures of periodontal disease—periodontal pocket depth (measures the distance between the gum tissue and its attachment to the tooth, loss of attachment (the distance between the point at which the gum is attached and the ‘neck’ of the tooth where the gum is attached in a healthy tooth), and bleeding on probing (a marker of current inflammation of the gums). Periodontal disease measurements were made in six index teeth (three in the upper arch and three in the lower arch), one per mouth sextant of the mouth. First molars were measured in the four posterior sextants, and right central incisors in the two anterior sextants; where the first molar was missing, the following tooth was chosen in order of priority: second premolar, first premolar, second molar; if the central incisor was missing, the next mesial tooth available in that sextant was chosen. Loss of attachment and gingival bleeding were assessed at two sites (mesiobuccal and distobuccal) on each index tooth, and periodontal pocket depth was measured on the mesiobuccal site. A Community Periodontal Index of Treatment Needs (CPITN) probe was used, with a 0.5 mm ball-ended tip with markings at 0 to 3.5, >3.5 to 5.5 and >5.5 mm. Examiners (research nurses) underwent extensive training and calibration including a pilot prior to the study and a calibration check during the study. Agreement was tested between each examiner and the training examiner (dental surgeon) for every reading for the three periodontal disease measures (loss of attachment, periodontal pocket depth, gingival bleeding). Examiner and trainer agreement ranged from 89% to 95% (closest agreement was for gingival bleeding), and the median κ index was 0.79. It was not possible to include other measures of dental disease (such as dental caries) in the dental examination because of time constraints on the physical examination of participants; the dental examination was part of an extensive physical examination. Given the advanced age of the participants (71–92 years), it was important to avoid participant burden and, therefore, only a very brief dental examination was possible.

The questionnaire included the following self-reported oral health measures: presence of teeth or dentures, self-rated oral health (excellent, good, fair, poor), experience of dental problems, oral impacts on daily life, dry mouth and dental service use (frequency of visiting a dentist and time since last dental visit). Participants were asked questions on common dental or oral health problems experienced in the past 6 months, including toothache or sensitivity, loose tooth or gum problems, bad position of teeth, fractured tooth, loose or ill-fitting dentures, appearance of teeth. Oral health-related quality of life (OHRQoL) was assessed through the Oral Impact on Daily Performances (OIDP) measure.^12^ Participants were asked whether in the past 6 months any oral health problems caused any of the following: difficulty eating, difficulty speaking, difficulty going out (eg, to shop or visit someone), difficulty relaxing (including sleeping), problems with smiling, laughing and showing teeth without embarrassment, emotional problems such as becoming more easily upset than usual, problems enjoying the company of others (eg, family, friends, neighbours). The Xerostomia Inventory (XI), a validated tool to assess dry mouth and its severity, was also used in the questionnaire.^13^ The XI questions include asking whether in the past 4 weeks the participants experienced the following symptoms: mouth feels dry, difficulty eating dry foods, getting up at night to drink, mouth feels dry when eating a meal, sip liquids to aid swallowing food, sucking sweets to relieve dry mouth, difficulties swallowing certain foods, skin of face feels dry, eyes feel dry, lips feel dry, inside of nose feels dry. Responses to each question were never, hardly ever, occasionally, fairly often or very often.

### Statistical analyses

Descriptive analyses were carried out to determine the prevalence of the different aspects of oral health measures. The number of teeth was categorised into no teeth, 1–7, >7–14, 15–20 and >21 teeth.^14^ Periodontal disease based on loss of attachment was categorised based on the proportion of sites with >5.5 mm; this was calculated as the number of sites affected with a loss of attachment of >5.5 mm as a proportion of sites examined, and categorised as 0%, 1–20% and >20% sites affected. For periodontal pockets, we calculated the number of sites with >3.5 mm pocket depth as a proportion of sites examined, in order to obtain the percentage of sites affected, which was further categorised into 0%, 1–20% and >20% sites affected. This approach has been used in previous epidemiological studies.^15^ ^16^ The number of teeth, periodontal disease prevalence and self-reported oral health conditions were examined to demographic characteristics (age, social class and region of residence). χ^2^ Tests were used to assess the statistical significance of the observed differences. Age was categorised into two groups of 71–79 and 80–92 years. Social class was used as two categories of non-manual and manual. Region was based on the town of residence and categorised into groups of the four British regions represented in the study—South of England, Wales/Midlands, North of England and Scotland. Analyses were carried out using SAS V.9.3.

## Results

A total of 1722 men (55% participation rate) attended the examination. Questionnaires were completed by 2147 men (68% response rate), including all those who attended the examination. Overall, the mean age of the study population was 78 years, and 47% were from manual social class. Compared with the men who responded to the questionnaire, the non-responders were older (mean age 80 years), and had a higher proportion of manual social classes (61%). Based on data from a previous follow-up, the non-responders also had higher levels of poor/fair self-rated health (27%) compared with responders (16%).

Of the 1722 men who were examined, 62 (3.6%) did not have information on objective clinical oral health; therefore, analyses based on these measures were restricted to 1660 men. Of those, 338 (20%) had no natural teeth, 728 (43%) had 1–20 teeth and 594 (36%) had ≥21 teeth. Table 1 presents the prevalence of edentulism, number of teeth, periodontal disease and gingival inflammation by age groups, social class and region. Overall, 20% men were edentulous. The prevalence of edentulism was greater in the older age group (80–92 years), and in those from manual social classes or from Scotland. Periodontal disease was measured in 1246 dentate men (those with natural teeth). Overall, 43% of men (n=536) had loss of attachment >5.5 mm; 24% (n=303) of men had >20% of sites affected by loss of attachment of 5.5 mm and 19% had 1–20% sites so affected. Overall, 44% of men had a periodontal pocket >3.5 mm. The proportion of men with 1–20% sites affected by periodontal pockets >3.5 mm was 15% (n=183) and that with >20% of sites was 29% (n=365). A small proportion (3%) had periodontal pockets >5.5 mm. Gingival bleeding was present in 198 men (16%). The older age group (80–92 years) and those from manual social classes had a higher prevalence of attachment loss and deeper pocket depth (>20% sites affected) than those aged 71–79 years or those of non-manual social classes, respectively. Those in the North of England had a lower prevalence of attachment loss and periodontal pockets than those from other regions. The prevalence of gingival bleeding did not differ by age, social class or region.

**Table 1:** Number of teeth and periodontal disease in a population-based study of 1660 older British men with dental examination aged 71–92 years in 2010–2012 in the British Regional Heart Study and sociodemographic factors

		Age groups		Social class		Region			
	n (%)	71–79 years (n=1107, 67%)	80–92 years (n=553, 33%)	Non-manual (n=861, 53%)	Manual (n=751, 47%)	South of England (n=589, 35%)	Wales/Midlands (n=264, 16%)	North of England (n=626, 38%)	Scotland (n=181, 11%)
Number of teeth in 1660 men									
No teeth	338 (20%)	183 (17%)	155 (28%)	123 (14%)	205 (27%)	82 (14%)	61 (23%)	137 (22%)	58 (32%)
1–7 teeth	123 (7%)	70 (6%)	53 (10%)	51 (6%)	70 (9%)	46 (8%)	19 (7%)	40 (6%)	18 (10%)
>7–14 teeth	265 (16%)	168 (15%)	97 (18%)	121 (14%)	134 (18%)	94 (16%)	43 (16%)	108 (17%)	20 (11%)
15–20	340 (20%)	233 (21%)	107 (19%)	185 (21%)	148 (20%)	119 (20%)	64 (24%)	116 (19%)	41 (23%)
≥21 teeth	594 (36%)	453 (41%)	141 (26%)	381 (44%)	194 (26%)	248 (42%)	77 (29%)	225 (36%)	44 (24%)
p Value		<0.0001		<0.0001		<0.0001			
Periodontal disease in 1246 dentate men									

		**Age groups**		**Social class**		**Region**			
	**n (%)**	**71–79 years (n=880, 71%)**	**80–92 years (n=366, 29%)**	**Non-manual (n=696, 57%)**	**Manual (n=519, 43%)**	**South of England (n=481, 39%)**	**Wales/ Midlands (n=192, 15%)**	**North of England (n=460, 37%)**	**Scotland (n=113, 9%)**

Periodontal disease based on per cent of sites with loss of attachment >5.5 mm									
0%	710 (57%)	520 (59%)	190 (52%)	401 (57%)	290 (56%)	262 (54%)	90 (47%)	293 (64%)	65 (58%)
1–20%	233 (19%)	167 (19%)	66 (18%)	146 (21%)	82 (16%)	91 (19%)	42 (22%)	83 (18%)	17 (15%)
>20%	303 (24%)	193 (22%)	110 (30%)	149 (21%)	147 (28%)	128 (27%)	60 (31%)	84 (18%)	31 (27%)
p Value	–	0.008		0.006		0.001			
Periodontal pocket depth—per cent of sites >3.5 mm pocket depth									
0%	697 (56%)	505 (57%)	192 (53%)	391 (56%)	285 (55%)	280 (58%)	71 (37%)	273 (59%)	73 (65%)
1–20%	183 (15%)	132 (15%)	51 (14%)	114 (16%)	66 (13%)	67 (14%)	37 (19%)	69 (15%)	10 (9%)
>20%	365 (29%)	243 (28%)	122 (33%)	191 (27%)	167 (32%)	134 (28%)	84 (44%)	118 (26%)	29 (26%)
p Value		0.12		0.08		<0.0001			
Gingival bleeding									
No gingival bleeding	1040 (84%)	740 (85%)	300 (82%)	578 (83%)	116 (17%)	402 (84%)	157 (82%)	386 (85%)	95 (84%)
Presence of gingival bleeding	198 (16%)	134 (15%)	64 (18%)	437 (85%)	76 (15%)	77 (16%)	34 (18%)	69 (15%)	18 (16%)
p Value		0.32		0.37		0.87			

Table 2 presents the prevalence of self-rated oral health and presence of natural teeth and dentures in the 2147 men with questionnaire data. Overall, 35% reported fair/poor oral health, and 19% reported no natural teeth and wearing dentures. The prevalence of reporting fair/poor self-rated oral health was higher in older men and those from manual social classes, but did not vary by region. The self-report of having no natural teeth and wearing dentures was higher in older participants, manual social classes and those from Scotland.

**Table 2:** Self-reported oral health in a population-based study of 2147 men aged 71–92 years in 2010–2012 in the British Regional Heart Study and sociodemographic factors

		Age groups		Social class		Region			
	n (%)	71–79 years (n=1373, 64%)	80–92 years (n=774, 36%)	Non-manual (n=1081, 52%)	Manual (n=1003, 48%)	South of England (n=736, 34%)	Wales/Midlands (n=327, 15%)	North of England (n=843, 39%)	Scotland (n=241, 11%)
Self-rated oral health									
Excellent	271 (13%)	177 (13%)	94 (13%)	155 (15%)	103 (11%)	88 (12%)	43 (14%)	98 (12%)	42 (18%)
Good	1049 (51%)	707 (54%)	342 (48%)	561 (54%)	455 (49%)	366 (52%)	160 (52%)	412 (52%)	111 (48%)
Fair	587 (29%)	364 (28%)	223 (31%)	272 (26%)	303 (32%)	208 (29%)	81 (26%)	237 (30%)	61 (27%)
Poor	132 (6%)	71 (5%)	61 (8%)	56 (5%)	72 (8%)	48 (7%)	24 (8%)	45 (6%)	15 (7%)
p Value		0.007		0.0003		0.43			
Natural teeth and dentures									
Only natural teeth	797 (38%)	562 (42%)	235 (31%)	467 (44%)	313 (32%)	292 (40%)	109 (34%)	318 (39%)	78 (33%)
Both natural teeth and dentures	882 (42%)	565 (42%)	317 (42%)	462 (44%)	390 (40%)	336 (47%)	133 (42%)	326 (39%)	87 (37%)
No natural teeth, and wear dentures	407 (19%)	210 (16%)	197 (26%)	128 (12%)	265 (27%)	92 (13%)	71 (22%)	175 (21%)	69 (29%)
Neither natural teeth nor dentures	14 (1%)	7 (1%)	7 (1%)	3 (0.28%)	10 (1%)	2 (0.28%)	3 (1%)	7 (1%)	2 (1%)
p Value		<0.0001		<0.0001		<0.0001			

Table 3 presents data on other self-reported oral health problems. Overall, 25% reported having had problems of toothache, sensitivity or tooth decay. The prevalence of one or more problems related to teeth (including toothache, sensitivity, loose tooth, ill-fitting denture) was 42%. Reporting one or more such problems was lower in manual social classes (p=0.007), but did not differ significantly by age (p=0.35) or by region (p=0.11). The prevalence of OIDP was 11% for difficulty eating food and 14% overall (at least one oral impact such as eating, speaking, and going out). The prevalence of one or more oral impacts was higher in older men (p=0.001), but did not differ significantly by social class (p=0.01) or region (p=0.18). Table 4 presents the XI data. The mean xerostomia score was 16 (SD 6). Overall, 34% reported that their mouth felt dry occasionally or more often. Some 31% reported 1–2 dry mouth symptoms, 20% reported 3–5 symptoms and 8% reported >5 symptoms. Combining self-reported oral health problems, oral impacts on daily activities, and dry mouth, the prevalence of one or more self-reported dental problems was 73%.

**Table 3:** Prevalence of self-reported dental problems in a population-based study of 2147 British men aged 71–92 years in 2010–2012 in the British Regional Heart Study and sociodemographic factors

		Age groups		Social class		Region			
	n (%)	71–79 years	80–92 years	Non-manual	Manual	South of England	Wales/Midlands	North of England	Scotland
Dental problems									
Toothache, sensitivity, decay	538 (25%)	359 (26%)	179 (23%)	290 (27%)	231 (23%)	200 (27%)	80 (24%)	201 (24%)	57 (24%)
Loose tooth, or gum problems	246 (11%)	171 (12%)	75 (10%)	132 (12%)	108 (11%)	95 (13%)	27 (8%)	103 (12%)	21 (9%)
Bad position of teeth	64 (3%)	43 (3%)	21 (3%)	33 (3%)	28 (3%)	23 (3%)	10 (3%)	24 (3%)	7 (3%)
Ill-fitting denture or fractured tooth	277 (13%)	166 (12%)	111 (14%)	153 (14%)	112 (11%)	100 (14%)	38 (12%)	102 (12%)	37 (15%)
One or more of the above problems	908 (42%)	591 (43%)	317 (41%)	486 (45%)	392 (39%)	338 (46%)	133 (41%)	340 (40%)	97 (40%)
Impact on daily life due to dental problems									
Difficulty eating food	231 (11%)	122 (9%)	109 (14%)	104 (10%)	118 (12%)	86 (12%)	31 (9%)	81 (10%)	33 (14%)
Difficulty speaking	67 (3%)	32 (2%)	35 (5%)	27 (3%)	38 (4%)	23 (3%)	10 (3%)	31 (4%)	3 (1%)
Difficulty going out (eg, to shop or visit someone)	29 (1%)	17 (1%)	12 (2%)	9 (1%)	18 (2%)	7 (1%)	6 (2%)	14 (2%)	2 (1%)
Difficulty relaxing (including sleeping)	31 (1%)	19 (1%)	12 (2%)	11 (1%)	20 (2%)	9 (1%)	4 (1%)	15 (2%)	3 (1%)
Problems with smiling, laughing without embarrassment	83 (4%)	49 (4%)	34 (4%)	40 (4%)	39 (4%)	25 (3%)	17 (5%)	32 (4%)	9 (4%)
Emotional problems (example, becoming more easily upset than usual)	26 (1%)	13 (1%)	13 (2%)	10 (1%)	16 (2%)	7 (1%)	5 (2%)	12 (1%)	2 (1%)
Problems enjoying the company of others (example, family, friends)	31 (1%)	17 (1%)	14 (2%)	14 (1%)	15 (2%)	10 (1%)	6 (2%)	12 (1%)	3 (1%)
One or more of the above problems	304 (14%)	169 (12%)	135 (17%)	140 (13%)	154 (15%)	104 (14%)	43 (13%)	112 (13%)	45 (19%)

**Table 4:** Xerostomia (dry mouth) Inventory in a population-based study of 2147 men aged 71–92 years in 2010–2012 in the British Regional Heart Study

	Never n (%)	Hardly ever n (%)	Occasionally n (%)	Fairly often n (%)	Very often n (%)
My mouth feels dry	963 (45)	449 (21)	516 (24)	149 (7)	70 (3)
I have difficulty in eating dry foods	1676 (78)	261 (12)	149 (7)	44 (2)	17 (1)
I get up at night to drink	1318 (61)	276 (13)	378 (18)	116 (5)	59 (3)
My mouth feels dry when eating a meal	1736 (81)	263 (12)	112 (5)	26 (1)	10 (0.50)
I sip liquids to aid in swallowing food	1687 (79)	192 (9)	187 (9)	55 (3)	26 (1)
I suck sweets or cough lollies to relieve dry mouth	1689 (79)	176 (8)	224 (10)	41 (2)	17 (1)
I have difficulties swallowing certain foods	1798 (84)	175 (8)	123 (6)	34 (2)	17 (1)
The skin of my face feels dry	1747 (81)	169 (8)	141 (7)	60 (3)	30 (1)
My eyes feel dry	1585 (74)	172 (8)	260 (12)	86 (4)	44 (2)
My lips feel dry	1480 (70)	241 (11)	321 (15)	81 (4)	24 (1)
The inside of my nose feels dry	1535 (72)	239 (11)	278 (13)	71 (3)	24 (1)

Based on questions on use of dental services, 11% reported going to a dentist only when having a problem, 15% reported that they had never gone to a dentist.

## Discussion

In this study, we aim to describe the burden of poor oral health in a socially representative sample of older British men aged 71–92 years. Our findings show a high burden of oral health problems including tooth loss, periodontal disease, poor self-rated oral health, oral impacts on eating and dry mouth. Several of these oral health conditions (including complete tooth loss, periodontal disease and poor self-rated oral health) were greater in lower social classes and the older age groups. These findings emphasise the high oral healthcare needs in older populations and the need to understand ways to prevent and manage these problems.

To our knowledge, this paper presents the most recent epidemiological population-based study of oral health and function in a community-dwelling older British population aged over 70 years with objective clinical and self-reported measures; there are few such data on older populations in the UK, apart from the 10 yearly Adult Dental Health Surveys which are conducted across the adult population, and the National Diet and Nutrition Survey from 1994 to 1995. Other studies in older populations in the UK have limited self-reported data on oral health.^8^ We present results from a cross-sectional study of a socially and geographically representative cohort of older British men. However, survivor bias is inevitable in a cohort sample of an ageing population; participants with higher rates of chronic diseases would have died. The moderate response rate for the clinical examination (55%) is also likely to have excluded participants in worse health. As observed in previous examinations,^17^ non-responders were older than responders and had higher proportions of manual social class and poor/fair self-rated overall health, which are also likely to be associated with having worse oral health. Therefore, it is possible that our findings have underestimated the burden of oral health in the older population. Another limitation is that this study comprises only white European men, and the findings cannot be generalised to women or other ethnic groups. The Adult Dental Health Survey reported better periodontal health in women than men.^6^ Nevertheless, we believe the findings provide a valuable insight into the epidemiology and burden of poor oral health in older British populations.

Reports from the 10 yearly Adult Dental Health Surveys (most recently in 2009) have shown that the prevalence of edentulism (no natural teeth) in adults declined by 22% from 1978 to 6% in 2009.^7^ ^18^ The proportion of adults with ≥21 teeth (widely used to define a minimum functional dentition) is reported to have increased from 73% in 1978 to 86% in 2009.^18^ These patterns have also been observed in countries other than the UK.^5^ In the National Diet and Nutrition Survey of 1994–1995, 45% of free-living adults aged >65 years were edentulous.^19^ Although retention of teeth has improved overall in adults, tooth loss (partial or complete) increases dramatically with age and remains a significant problem in older age.^18^ The Adult Dental Health Survey 2009 documented the marked increase in loss of teeth in older age groups;^18^ the prevalence of edentulism was 30% in adults aged 75–84 years and 47% in those aged >85 years. In the English Longitudinal Study of Ageing, 26% of men and women >60 years in 2002–2003 reported having no natural teeth.^20^ Our findings, based on men aged 71–92 years, showed a prevalence of being edentate of 20%, and that only 31% had ≥21 teeth.

We used three measures to assess periodontal disease—gingival bleeding (a marker of current inflammation of the gums), periodontal pockets (a deeper pocket indicates active periodontal disease) and loss of attachment (a marker of experience of periodontal disease).^6^ Our sample of older men had a high prevalence of excess loss of attachment, a longer term measure of damage to periodontal tissue, while deep periodontal pockets and gingival inflammation (indicators of active periodontal disease) were less prevalent. The Adult Dental Health Survey reported higher proportions of severe (76%) and moderate loss of attachment (25%) than our study^21^; this could be higher since it was based on the highest measure recorded on any tooth, whereas our measure was based only on six index teeth. Apart from the Adult Dental Health Survey, most studies on the prevalence of periodontal disease and oral disease in older people are from non-UK populations. Variations in measurement of periodontal disease make it difficult for comparisons between studies, with few national level data on periodontal disease;^5^ estimates of periodontal disease prevalence in 65–74-year olds are reported to range from 4% in New Zealand to 40% in Germany.^5^

Self-reported oral health problems in our study ranged from self-rated oral health to dry mouth symptoms. We found that over a third of the participants reported fair or poor self-rated oral health. The most prevalent oral health problems were toothache, sensitivity and ill-fitting dentures. Over 40% of men reported one or more oral health problems. The most common oral impact was difficulty eating. In relation to xerostomia, a third of participants reported that their mouth felt dry, and a third reported one or two symptoms of dry mouth. Over 70% of our sample reported one or more of these problems combined (problems with teeth or gums, oral impact on daily activities and dry mouth). Notably, our findings also show a very high prevalence (73%) of oral health problems occurring in combination, such as problems with teeth/gums along with difficulty eating and dry mouth. The Adult Dental Health Survey also reported high rates of self-reported oral health problems such as impact on eating, particularly in older age groups.^22^

The prevalence of most oral health conditions (edentulism, lower number of teeth, severe periodontal disease and fair/poor self-rated oral health) was greater in manual (or lower socioeconomic groups) than in non-manual social class groups in our study. Similar patterns have been reported in other British studies.^8^ ^23^ Determinants of these socioeconomic differences in oral health in older populations need to be further investigated. Regional differences were most markedly observed for edentulism, with the lowest rates in the South of England and the highest in Scotland. This is in keeping with observations of other conditions such as cardiovascular disease.^24^

## Implications and conclusions

This study highlights a substantial burden of oral health in the older population which has important implications for public health policy, clinical practice and research. Improving the health of an increasingly ageing population needs to address the oral health problems in this population, particularly in those from lower socioeconomic status groups. Treatment and management of oral health problems in older people is further complicated by age-related changes in the mouth, the presence of comorbidities and issues of access to dental care.^25^ ^26^ Care pathways for oral healthcare of older people need to adapt to the needs of older people.^27^ Ageing research also currently largely focuses on managing long-term conditions and improving disability and frailty in older age; there is little emphasis on preventing oral health problems in later life and its importance in improving healthy ageing. There remains a need to investigate determinants (biological and social) that are important in improving oral health and function in later life. Population-based studies are also needed to understand the contribution of oral health to overall health in later life alongside other aspects of healthy ageing such as disability, frailty and chronic diseases.
